# Supplementary material for: Are Mediterranean Island Mountains Hotspots of Taxonomic and Phylogenetic Biodiversity? The Case of the Endemic Flora of the Balearic Islands
Source: Plants (Basel). 2023 Jul 13;12(14):2640. doi: 10.3390/plants12142640 (PMC10386412; doi:10.3390/plants12142640)
Supplement: Supplementary file 1 [file plants-12-02640-s001.zip › plants-2484991-supplementary/Guardiola&Sáez_2023_Supplementary.docx]

**Are Mediterranean island mountains hotspots of taxonomic and phylogenetic biodiversity? The case of the endemic flora of the Balearic Islands**

Moisès Guardiola^a*^ & Llorenç Sáez^b,c^

^a^ Unit of Botany, Department of Animal and Plant Biology and Ecology, Universitat Autònoma de Barcelona, E-08193 Bellaterra, Spain

^b^ Systematics and Evolution of Vascular Plants (UAB) – Associated Unit to CSIC by IBB, Unit of Botany, Department of Animal and Plant Biology and Ecology, Universitat Autònoma de Barcelona, ES-08193 Bellaterra, Spain

^c^ Societat d’Història Natural de les Balears (SHNB), Margarida Xirgu 16, ES-07003 Palma de Mallorca, Spain

**E-mails of all authors:** Moisès Guardiola: Moises.Guardiola@uab.cat, Llorenç Sáez: gymnesicum@yahoo.es

**Supplementary Materials**

**Figure S1**: Map of the Balearic Islands with main geographical names mentioned in this study and the limits of the Natura 2000 network (blue lines).


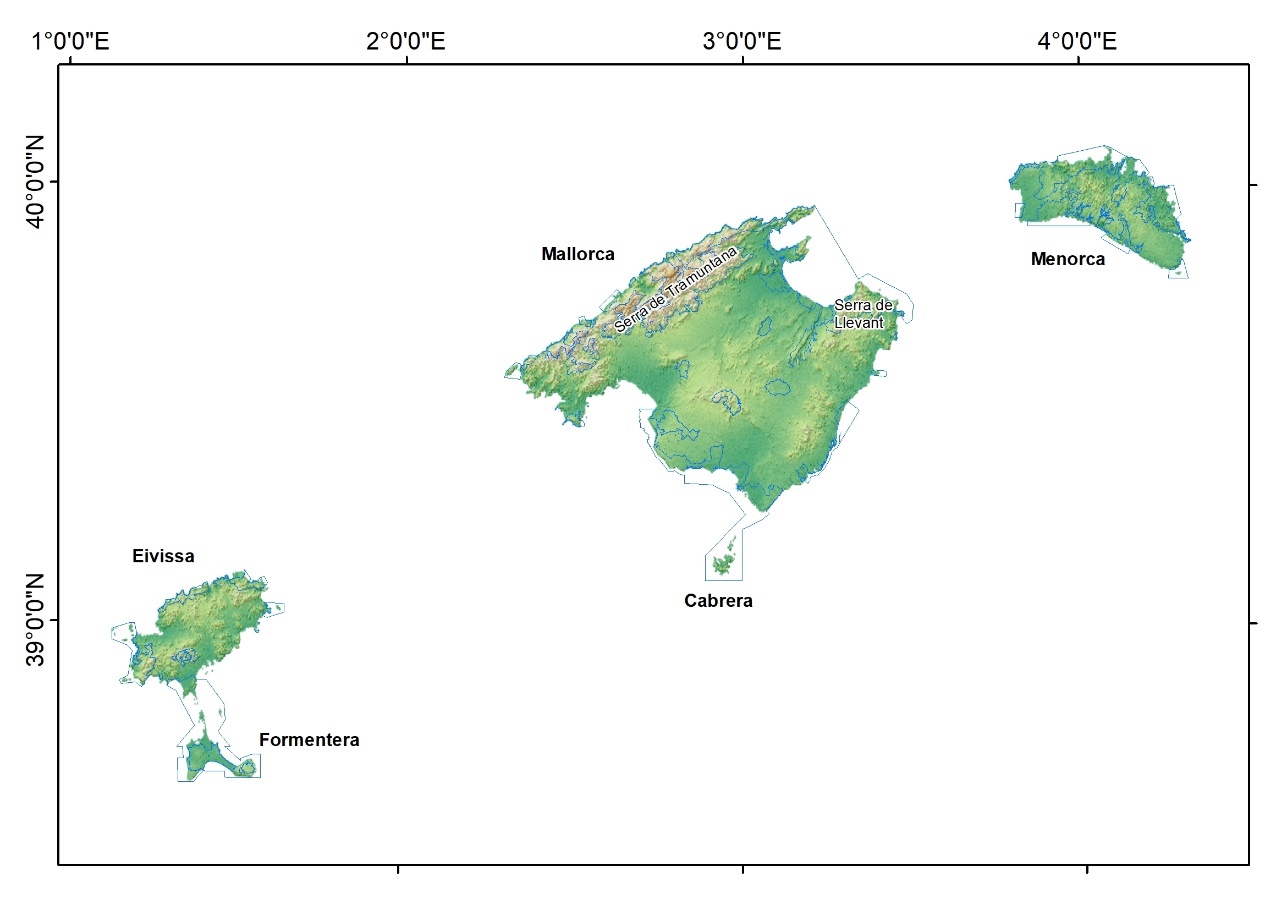


**Figure S2**: Maps of the Balearic Islands showing the spatial distribution of (a) elevation, (b) aspect, (c) slope, (d) distance to coast, (e) annual mean temperature (Bio1), (f) mean temperature of wettest quarter (Bio8) and (g) annual precipitation (Bio12).

| 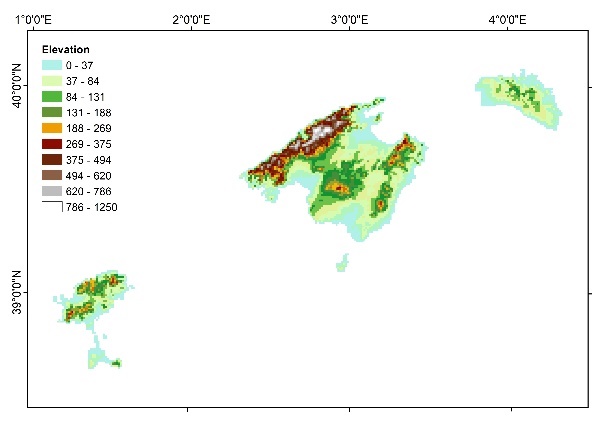 | 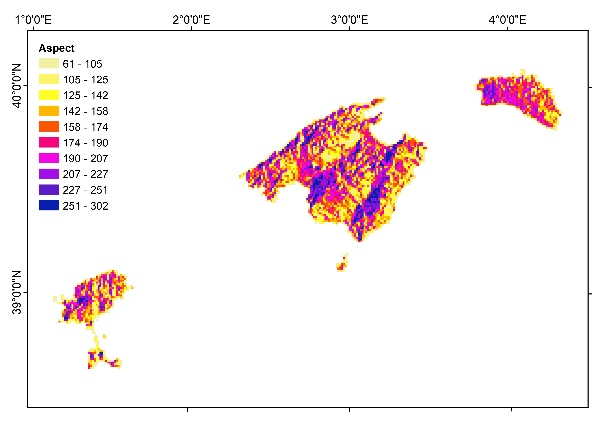 |
| --- | --- |
| 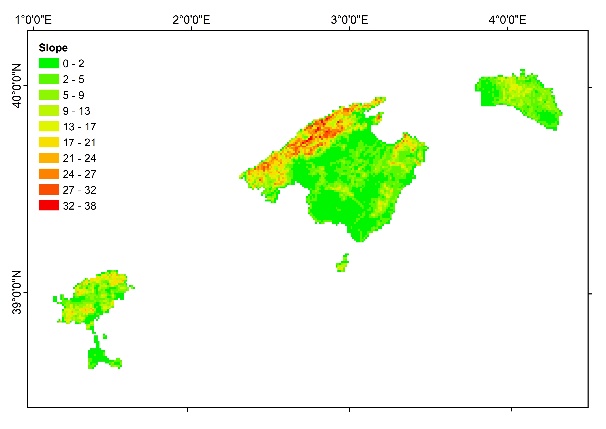 | 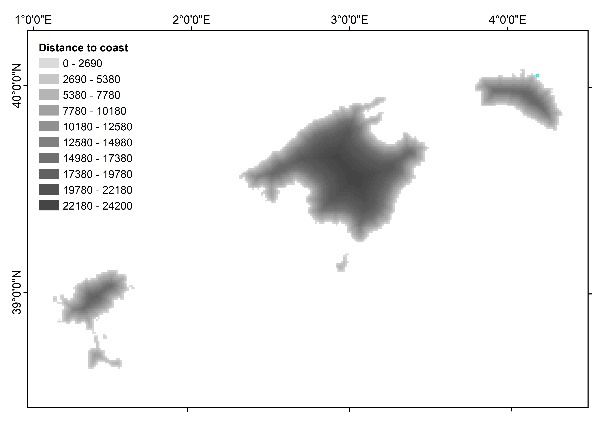 |
| 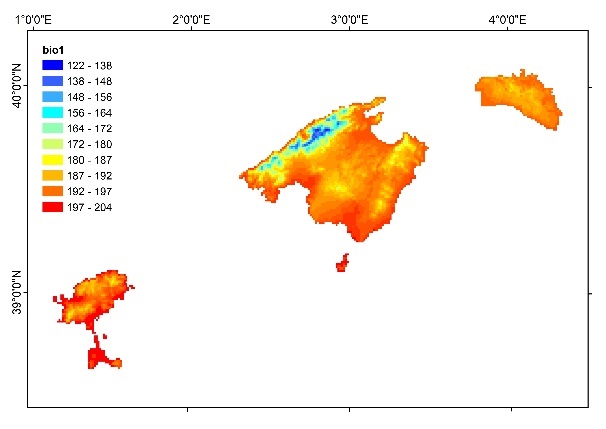 | 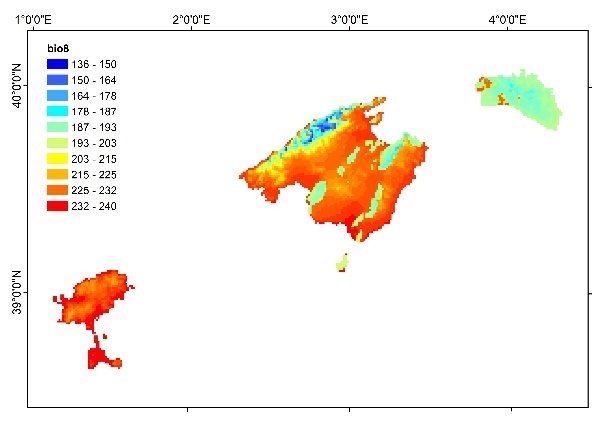 |
| 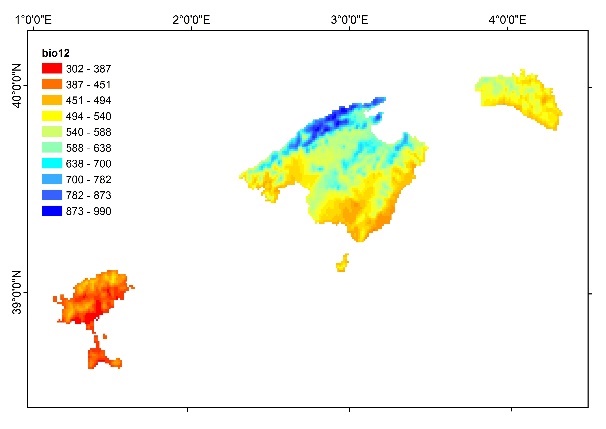 |  |

**Figure S3**: Correlation plot of all studied species richness and phylogenetic diversity metrics.


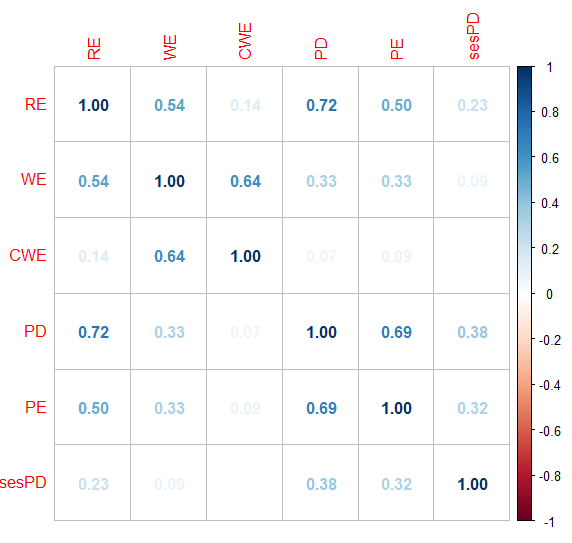


**Figure S4**: Maps showing: (a) endemic species richness (RE), (b) weighted endemism (WE), (c) corrected weighted endemism (CWE), (d) phylogenetic diversity (PD), (e) standardised effect size for phylogenetic diversity (sesPD) and (f) phylogenetic endemism (PE).

| 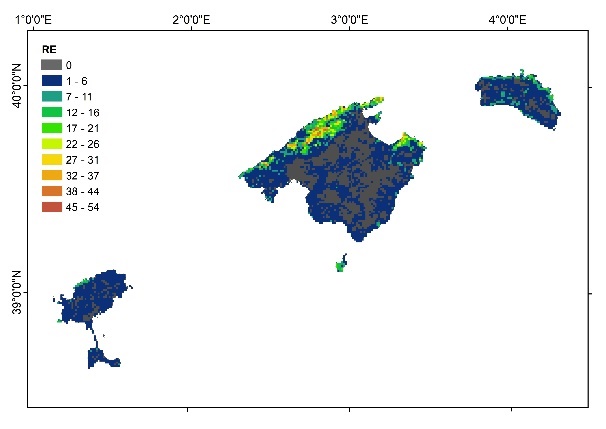 | 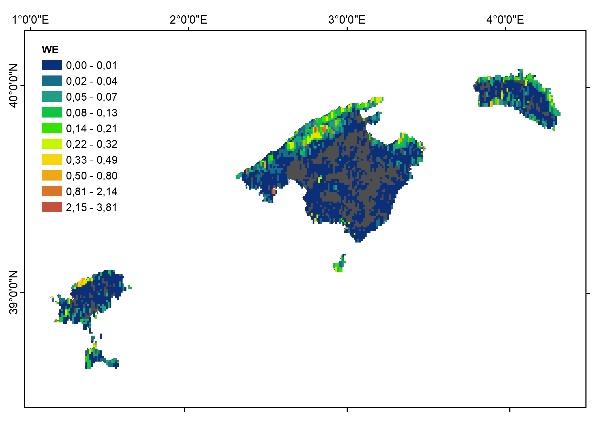 |
| --- | --- |
| 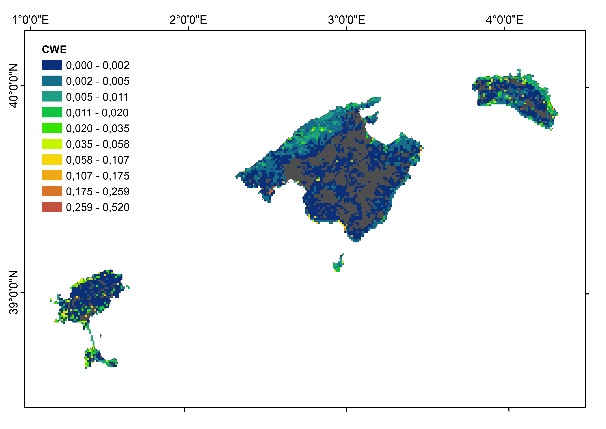 | 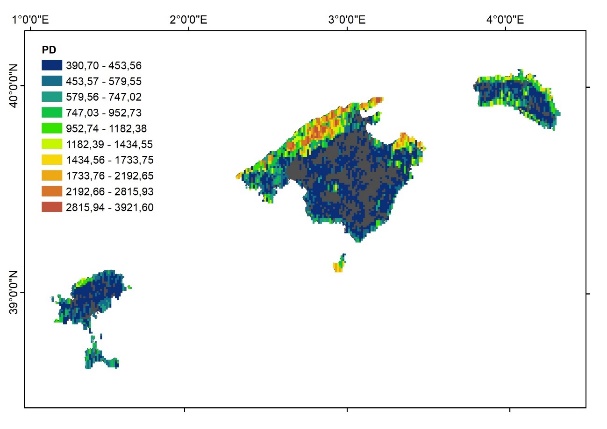 |
| 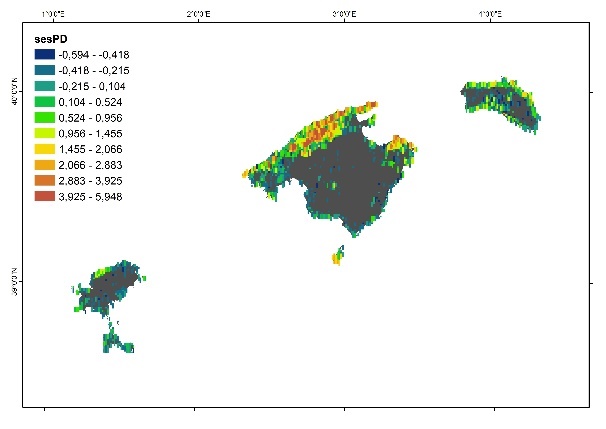 | 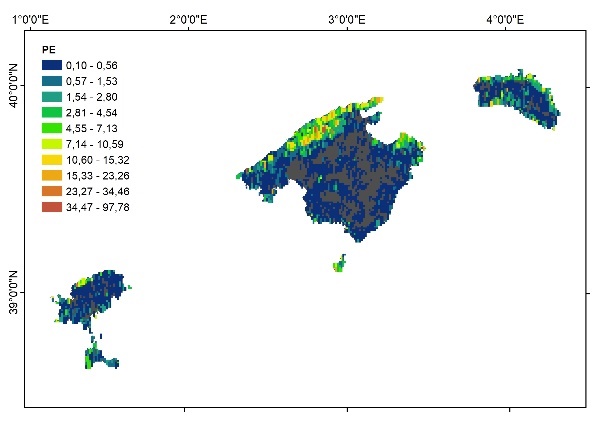 |

**Figure S5**: Spatial distribution of the squares with significant high sesPD or phylogenetic overdispersion (red) and squares with significant low sesPD or phylogenetic clustering (blue).


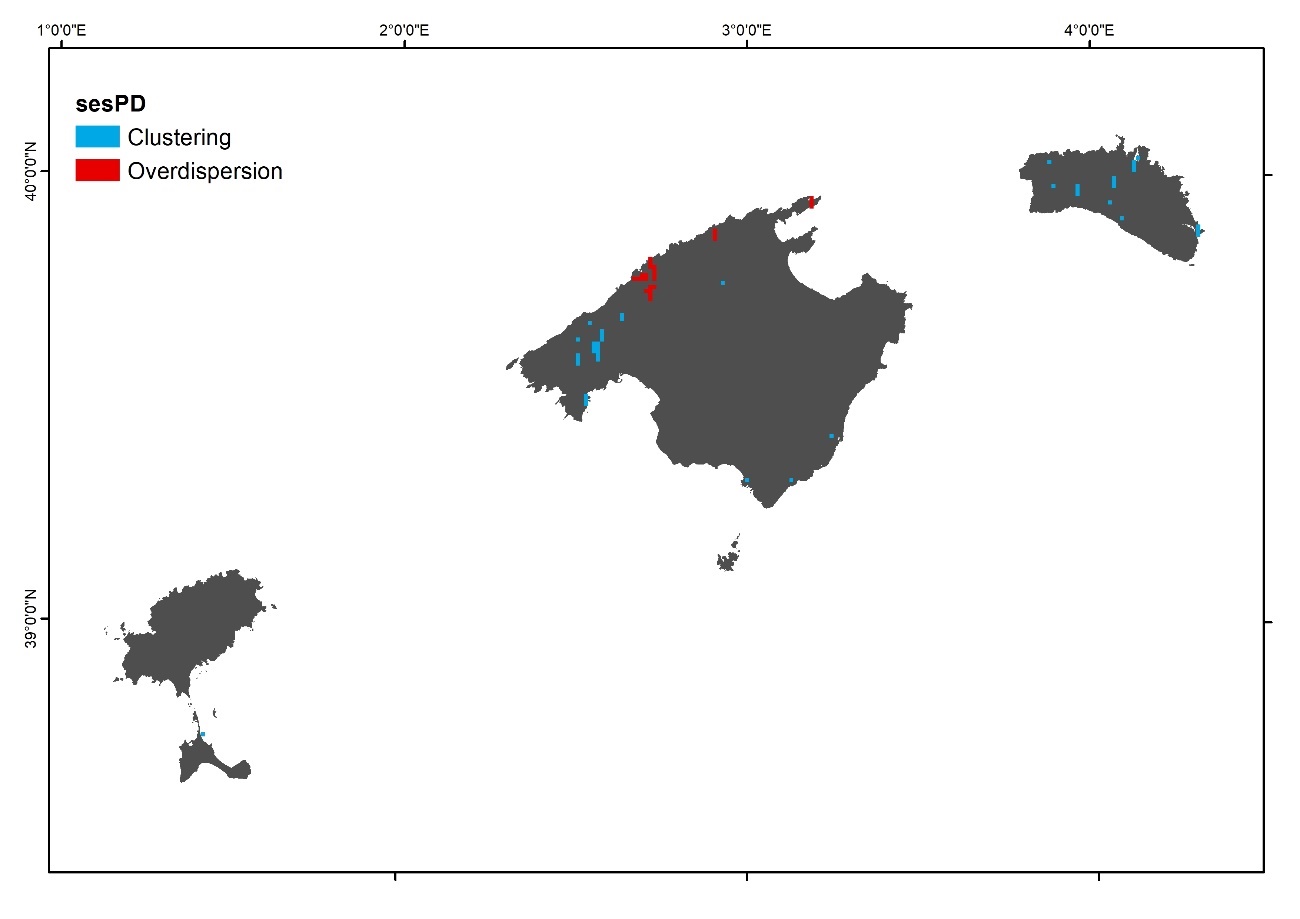


**Figure S6**: Spatial distribution of the (a) sum of the EDGE values of each species at each UTM square and (b) top richest (1%, 2.5%, 5% and 10%) grid cells of the EDGE values.

| 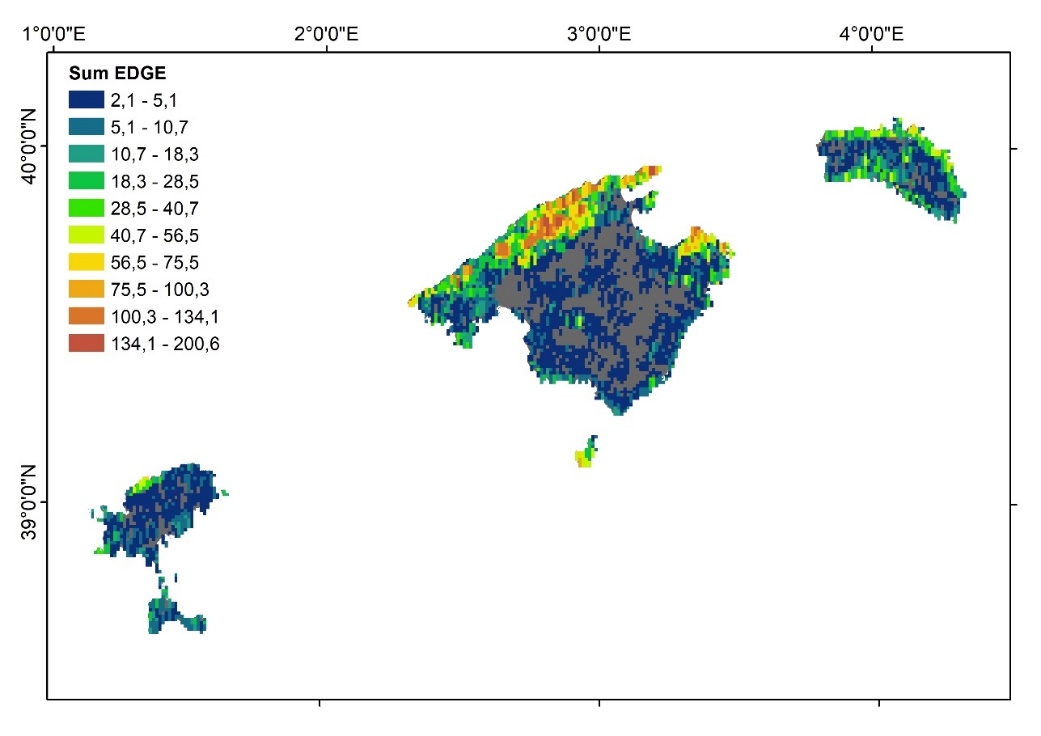 |
| --- |
| 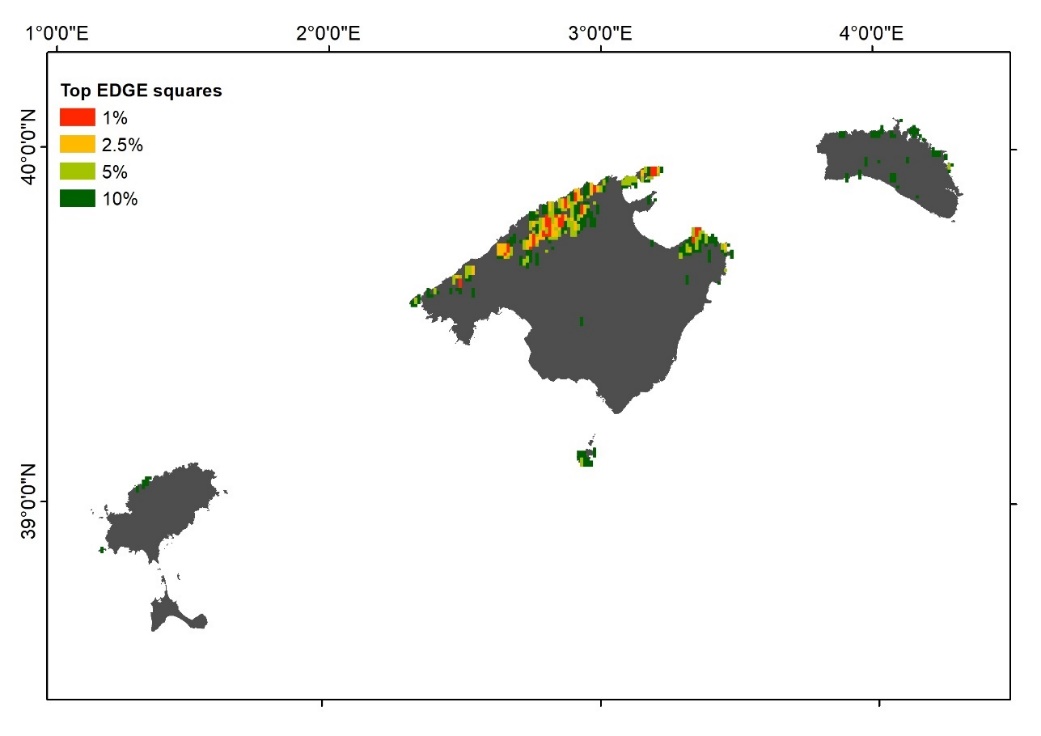 |

**Figure S7**: Maps showing top richest (1%, 2.5%, 5% and 10%) grid cells of (a) endemic species richness (RE), (b) weighted endemism (WE), (c) corrected weighted endemism (CWE), (d) phylogenetic diversity (PD), (e) standardised effect size for phylogenetic diversity (sesPD) and (f) phylogenetic endemism (PE).

| 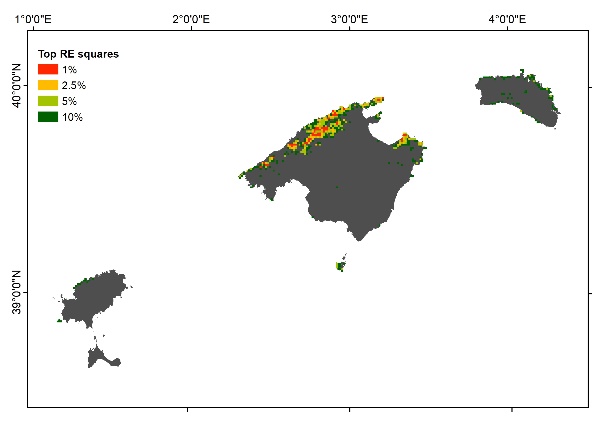 | 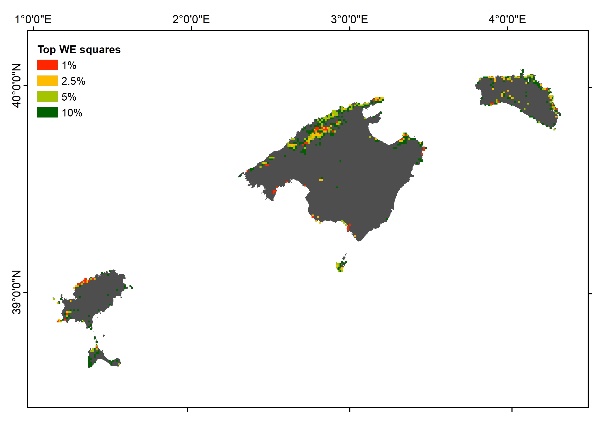 |
| --- | --- |
| 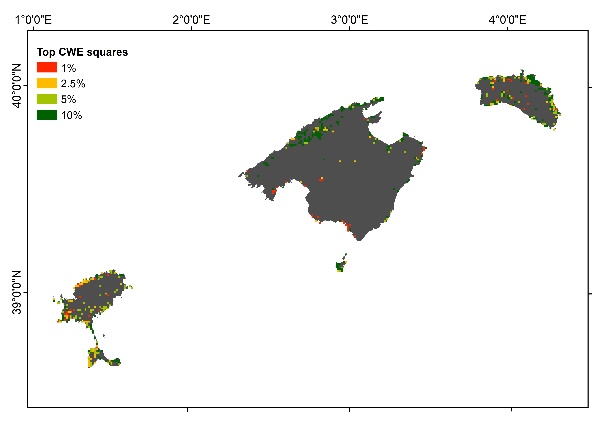 | 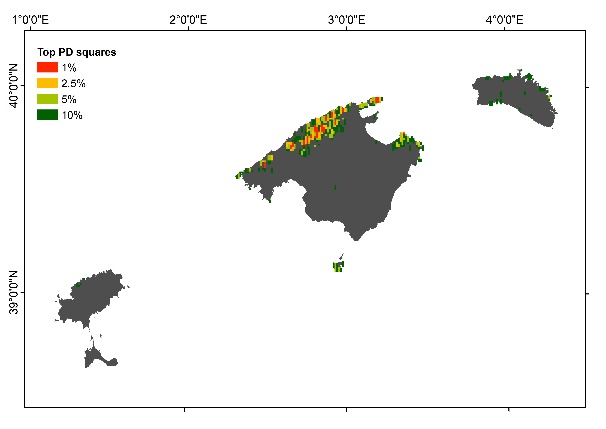 |
| 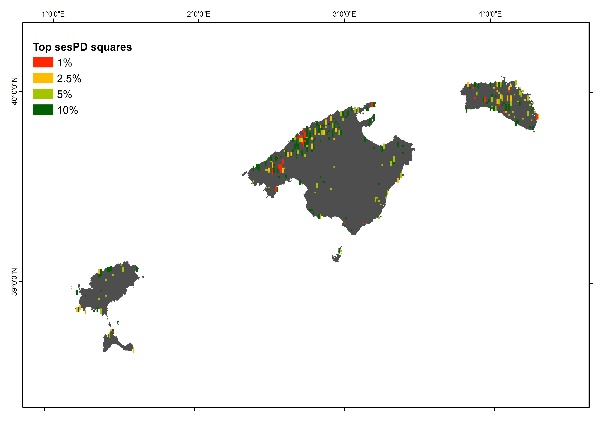 | 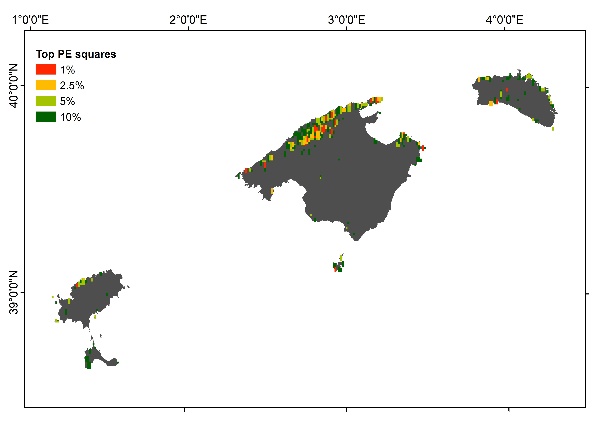 |

**Figure S8**: Spatial distribution of the percentage of each species' habitat (pie charts) in each 1x1 km UTM square.


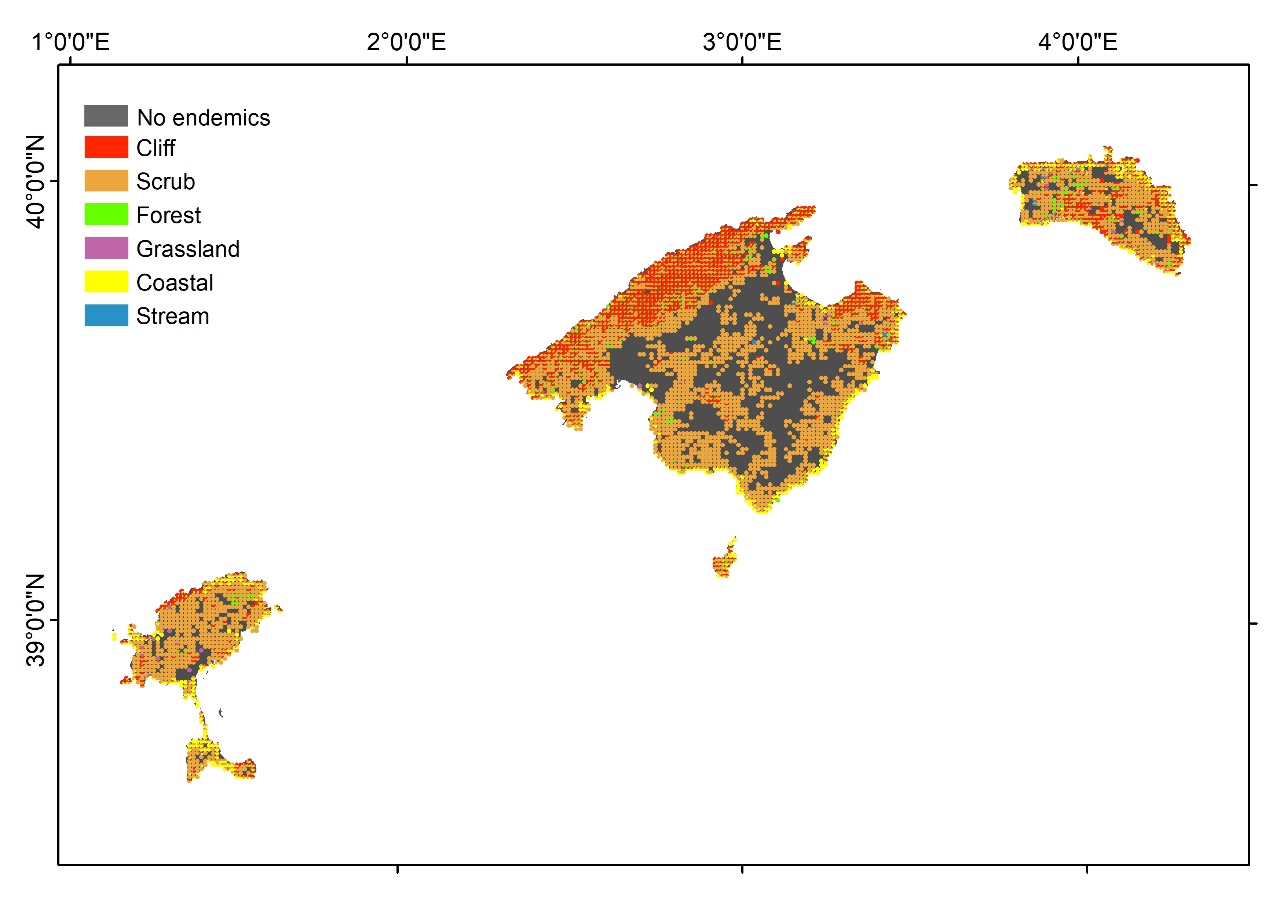


**File S1**: Phylogeny_Endemics_Balearic_Islands.pdf. Dated tree of the endemic flora of the Balearic Islands.

**Table S1**: List of plant species used in our analysis, number of 1x1 km squares were appears, main habitat where grows, species IUCN red list category (according to Sáez et al. 2017 and unpublished data): LC = least concern, NT = near threatened, VU = vulnerable, EN = endangered, CR = critically endangered, DD = data deficient, and NE = not evaluated by under IUCN criterion; type of endemism (endemic or subendemic) and EDGE value.

| **Species** | **Family** | **Number 1 km^2^ squares** | **Habitat** | **IUCN** | **Endemism** | **EDGE** |
| --- | --- | --- | --- | --- | --- | --- |
| *Agrostis barceloi* L. Sáez & Rosselló | Poaceae | 1 | Rocky | CR | Endemic | 4,371 |
| *Aira minoricensis* Fraga, Romero-Zarco & L. Sáez | Poaceae | 5 | Grassland | LC | Endemic | 3,673 |
| *Allium antonii-bolosii* P. Palau subsp. *antonii-bolosii* | Amaryllidaceae | 50 | Rocky | LC | Endemic | 3,910 |
| *Allium antonii-bolosii* subsp. *eivissanum* (Garbari & Miceli) N. Torres & Rosselló | Amaryllidaceae | 22 | Rocky | LC | Endemic | 3,910 |
| *Allium grosii* Font Quer | Amaryllidaceae | 19 | Rocky | LC | Endemic | 3,910 |
| *Anthyllis hystrix* (Barceló) Cardona, Contandr. & Sierra | Fabaceae | 62 | Littoral | NT | Endemic | 3,219 |
| *Anthyllis vulneraria* subsp. *balearica* (Marès & Vigin.) O. Bolòs & Vigo | Fabaceae | 39 | Rocky | LC | Endemic | 3,213 |
| *Arenaria bolosii* (Cañig.) L. Sáez & Rosselló | Caryophyllaceae | 3 | Scrub | VU | Endemic | 4,003 |
| *Arenaria grandiflora* subsp. *glabrescens* (Willk.) G. López & Nieto Feliner | Caryophyllaceae | 22 | Rocky | LC | Endemic | 3,934 |
| *Aristolochia bianorii* Sennen & Pau | Aristolochiaceae | 72 | Rocky | LC | Endemic | 4,932 |
| *Arum pictum* subsp. *sagittifolium* Rosselló & L. Sáez | Araceae | 391 | Scrub | LC | Endemic | 4,897 |
| *Asperula paui* Font Quer subsp. *paui* | Rubiaceae | 31 | Rocky | NT | Endemic | 3,204 |
| *Asplenium majoricum* Litard. | Aspleniaceae | 42 | Rocky | NT | Subendemic | 5,494 |
| *Astragalus balearicus* Chater | Fabaceae | 363 | Scrub | LC | Endemic | 3,765 |
| *Avellinia longiaristata* (Font Quer) Romero Zarco & L. Sáez | Poaceae | 3 | Grassland | NT | Endemic | 4,175 |
| *Bellium artrutxensis* P. Fraga & Rosselló | Asteraceae | 4 | Grassland | NT | Endemic | 3,340 |
| *Beta maritima* subsp. *marcosii* (O. Bolòs & Vigo) A. Juan & M.B. Crespo | Amaranthaceae | 9 | Littoral | VU | Endemic | 4,413 |
| *Biscutella ebusitana* Rosselló, N. Torres & L. Sáez | Brassicaceae | 27 | Rocky | NT | Endemic | 4,024 |
| *Brassica balearica* Pers. | Brassicaceae | 139 | Rocky | LC | Endemic | 3,975 |
| *Brimeura duvigneaudii* (L. Llorens) Rosselló, Mus & Mayol subsp. *duvigneaudii* | Asparagaceae | 9 | Rocky | VU | Endemic | 4,002 |
| *Brimeura duvigneaudii* subsp. *occultata* L. Sáez, Rita, Bibiloni, Roquet & López-Alvarado | Asparagaceae | 1 | Rocky | CR | Endemic | 4,625 |
| *Bupleurum barceloi* Willk. | Apiaceae | 132 | Rocky | LC | Endemic | 3,738 |
| *Carduncellus dianius* Webb | Asteraceae | 13 | Littoral | VU | Subendemic | 3,179 |
| *Carduus ibizensis* (Devesa & Talavera) Rosselló & N. Torres | Asteraceae | 12 | Grassland | LC | Endemic | 3,276 |
| *Carex rorulenta* Porta | Cyperaceae | 302 | Rocky | LC | Endemic | 4,620 |
| *Cephalaria squamiflora* subsp. *balearica* (Willk.) Greuter | Caprifoliaceae | 145 | Rocky | LC | Endemic | 3,828 |
| *Cephalaria squamiflora* subsp. *ebusitana* (O. Bolòs & Vigo) O. Bolòs | Caprifoliaceae | 3 | Rocky | EN | Endemic | 4,291 |
| *Chaenorhinum formenterae* Gand. | Plantaginaceae | 28 | Grassland | NT | Endemic | 3,565 |
| *Chaenorhinum rodriguezii* (Porta) L. Sáez & Vicens | Plantaginaceae | 6 | Rocky | EN | Endemic | 4,022 |
| *Clinopodium rouyanum* (Briq.) Govaerts | Lamiaceae | 11 | Rocky | NT | Endemic | 3,026 |
| *Coristospermum huteri* (Porta) L. Sáez & Rosselló | Apiaceae | 3 | Rocky | CR | Endemic | 3,901 |
| *Coronilla montserratii* Fraga & Rosselló | Fabaceae | 10 | Grassland | NT | Endemic | 3,498 |
| *Cotoneaster majoricensis* L. Sáez & Rosselló | Rosaceae | 3 | Rocky | CR | Endemic | 5,352 |
| *Crepis triasii* (Cambess.) Fries | Asteraceae | 427 | Rocky | LC | Endemic | 3,097 |
| *Crocus cambessedesii* J. Gay | Iridaceae | 444 | Rocky | LC | Endemic | 4,040 |
| *Cyclamen balearicum* Willk. | Primulaceae | 788 | Forest | LC | Subendemic | 4,375 |
| *Cymbalaria fragilis* (J.J. Rodr.) Chevalier | Plantaginaceae | 15 | Rocky | NT | Endemic | 3,592 |
| *Dactylis ibizensis* Gand. | Poaceae | 8 | Littoral | LC | Endemic | 3,673 |
| *Daphne rodriguezii* Teixidor | Thymelaeaceae | 40 | Scrub | VU | Endemic | 4,024 |
| *Delphinium pentagynum* subsp. *formenteranum* N. Torres, L. Sáez, Rosselló & C. Blanché | Ranunculaceae | 12 | Scrub | VU | Endemic | 4,274 |
| *Dianthus rupicola* subsp. *bocchoriana* L. Llorens & Gradaille | Caryophyllaceae | 13 | Rocky | NT | Endemic | 3,989 |
| *Digitalis minor* L. | Plantaginaceae | 350 | Rocky | LC | Endemic | 3,830 |
| *Diplotaxis ibicensis* (Pau) Gómez-Campo | Brassicaceae | 78 | Littoral | LC | Subendemic | 3,975 |
| *Dryopteris pallida* subsp. *balearica* (Litard.) Fraser-Jenk. | Dryopteridaceae | 85 | Rocky | LC | Endemic | 5,488 |
| *Erodium reichardii* (Murray) DC. | Geraniaceae | 59 | Rocky | LC | Endemic | 4,789 |
| *Euphorbia fontqueriana* Greuter | Euphorbiaceae | 2 | Scrub | EN | Endemic | 4,214 |
| *Euphorbia maresii* Knoche subsp. *maresii* | Euphorbiaceae | 16 | Scrub | VU | Endemic | 3,819 |
| *Euphorbia maresii* subsp. *balearica* (Willk.) Molero, Mus, Rosselló & Vallès-Xirau | Euphorbiaceae | 70 | Rocky | LC | Endemic | 3,751 |
| *Euphorbia margalidiana* Kuhbier & Lewej. | Euphorbiaceae | 1 | Littoral | VU | Endemic | 3,819 |
| *Euphorbia nurae* Fraga & Rosselló | Euphorbiaceae | 25 | Grassland | NT | Endemic | 3,757 |
| *Femeniasia balearica* (Rodr.) Susanna | Asteraceae | 9 | Littoral | VU | Endemic | 3,179 |
| *Filago petro-ianii* Rita & Dittrich | Asteraceae | 4 | Grassland | VU | Endemic | 3,081 |
| *Galium balearicum* Briq. | Rubiaceae | 47 | Rocky | LC | Endemic | 3,185 |
| *Galium crespianum* J.J. Rodr. | Rubiaceae | 271 | Rocky | LC | Endemic | 3,185 |
| *Galium friedrichi* N. Torres, L. Sáez, Mus & Rosselló | Rubiaceae | 41 | Rocky | LC | Endemic | 3,185 |
| *Genista dorycnifolia* Font Quer subsp. *dorycnifolia* | Fabaceae | 25 | Scrub | NT | Endemic | 3,465 |
| *Genista dorycnifolia* subsp. *grosii* (Font Quer) Font Quer & Rothm. | Fabaceae | 10 | Rocky | EN | Endemic | 3,923 |
| *Genista majorica* Cantó & M.J. Sánchez | Fabaceae | 266 | Rocky | LC | Endemic | 3,459 |
| *Genista valdes-bermejoi* Talavera & L. Sáez | Fabaceae | 20 | Scrub | VU | Endemic | 3,528 |
| *Globularia majoricensis* Gand. | Plantaginaceae | 207 | Rocky | LC | Endemic | 3,825 |
| *Helianthemum scopulicolum* L. Sáez, Alomar & Rosselló | Cistaceae | 1 | Rocky | CR | Endemic | 5,083 |
| *Helichrysum crassifolium* (L.) Loudon | Asteraceae | 489 | Rocky | LC | Endemic | 2,834 |
| *Helichrysum massanellanum* Herrando, J.M. Blanco, L. Sáez & Galbany | Asteraceae | 13 | Scrub | VU | Endemic | 2,903 |
| *Helictotrichon crassifolium* (Font Quer) Röser | Poaceae | 13 | Rocky | VU | Endemic | 3,748 |
| *Helosciadium bermejoi* (L. Llorens) Popper & M.F. Watson | Apiaceae | 1 | Stream | EN | Endemic | 4,614 |
| *Helleborus lividus* Aiton | Ranunculaceae | 52 | Rocky | NT | Endemic | 4,199 |
| *Hieracium balearicum* Arv.-Touv. | Asteraceae | 17 | Rocky | LC | Endemic | 2,952 |
| *Hieracium majoricanum* Arv.-Touv. | Asteraceae | 6 | Rocky | NT | Endemic | 2,959 |
| *Hippocrepis balearica* Jacq. | Fabaceae | 486 | Rocky | LC | Endemic | 3,198 |
| *Hipocrepis grosii* (Pau) Boira, Gil & L. Llorens | Fabaceae | 17 | Rocky | VU | Endemic | 3,267 |
| *Hypericum balearicum* L. | Hypericaceae | 535 | Scrub | LC | Endemic | 4,113 |
| *Hypericum hircinum* subsp. *cambessedesii* (Barceló) Sauvage | Hypericaceae | 16 | Stream | VU | Endemic | 4,181 |
| *Laserpitium gallicum* subsp. *majoricum* Romo | Apiaceae | 56 | Rocky | NT | Endemic | 3,395 |
| *Launaea cervicornis* (Boiss.) Font Quer & Rothm. | Asteraceae | 340 | Littoral | LC | Endemic | 3,016 |
| *Limonium alcudianum* Erben | Plumbaginaceae | 7 | Littoral | LC | Endemic | 2,115 |
| *Limonium antoni-llorensii* L. Llorens | Plumbaginaceae | 4 | Littoral | EN | Endemic | 3,522 |
| *Limonium barceloi* Gil & L. Llorens | Plumbaginaceae | 1 | Littoral | CR | Endemic | 3,043 |
| *Limonium bianorii* (Sennen & Pau) Erben | Plumbaginaceae | 7 | Littoral | LC | Endemic | 2,108 |
| *Limonium biflorum* (Pignatti) Pignatti | Plumbaginaceae | 40 | Littoral | LC | Endemic | 2,107 |
| *Limonium boirae* L. Llorens & Tébar | Plumbaginaceae | 2 | Littoral | CR | Endemic | 2,807 |
| *Limonium bolosii* Gil & L. Llorens | Plumbaginaceae | 1 | Littoral | DD | Endemic |  |
| *Limonium carvalhoi* Rosselló & L. Sáez | Plumbaginaceae | 1 | Littoral | CR | Endemic | 3,750 |
| *Limonium ejulabilis* Rosselló, Mus & Soler | Plumbaginaceae | 1 | Littoral | CR | Endemic | 2,916 |
| *Limonium escarrei* L. Llorens & Tébar | Plumbaginaceae | 1 | Littoral | DD | Endemic |  |
| *Limonium fontqueri* (Pau) Erben | Plumbaginaceae | 21 | Littoral | VU | Endemic | 2,175 |
| *Limonium formenterae* L. Llorens | Plumbaginaceae | 9 | Littoral | VU | Endemic | 3,127 |
| *Limonium grosii* L. Llorens | Plumbaginaceae | 24 | Littoral | VU | Endemic | 3,127 |
| *Limonium inexpectans* L. Sáez & Rosselló | Plumbaginaceae | 2 | Littoral | CR | Endemic | 2,852 |
| *Limonium leonardi-llorensii* L. Sáez, Carvalho & Rosselló | Plumbaginaceae | 2 | Littoral | CR | Endemic | 3,750 |
| *Limonium magallufianum* L. Llorens | Plumbaginaceae | 2 | Littoral | CR | Endemic | 2,916 |
| *Limonium majoricum* Pignatti | Plumbaginaceae | 4 | Littoral | VU | Endemic | 3,127 |
| *Limonium marisolis* L. Llorens | Plumbaginaceae | 3 | Littoral | VU | Endemic | 3,127 |
| *Limonium migjornense* L. Llorens | Plumbaginaceae | 3 | Littoral | CR | Endemic | 3,750 |
| *Limonium minoricense* Erben | Plumbaginaceae | 9 | Littoral | LC | Endemic | 2,107 |
| *Limonium minutum* (L.) Chaz. | Plumbaginaceae | 660 | Littoral | LC | Endemic | 2,107 |
| *Limonium pseudodyctyocladon* L. Llorens | Plumbaginaceae | 1 | Littoral | CR | Endemic | 3,750 |
| *Limonium saxicolum* Erben | Plumbaginaceae | 4 | Littoral | LC | Endemic | 2,115 |
| *Limonium wiedmannii* Erben | Plumbaginaceae | 3 | Littoral | VU | Endemic | 2,320 |
| *Linaria aeruginea* subsp. *pruinosa* (Sennen & Pau) Chater & Valdés | Plantaginaceae | 92 | Rocky | LC | Endemic | 3,559 |
| *Lonicera pyrenaica* subsp. *majoricensis* (Gand.) Gand. | Caprifoliaceae | 34 | Rocky | NT | Endemic | 4,204 |
| *Lotus fulgurans* (Porta) D.D. Sokoloff | Fabaceae | 55 | Littoral | NT | Endemic | 3,422 |
| *Lotus tetraphyllus* L. | Fabaceae | 308 | Rocky | LC | Endemic | 3,442 |
| *Magydaris pastinacea* subsp. *femeniesii* O. Bolòs & Vigo | Apiaceae | 5 | Grassland | DD | Endemic |  |
| *Malva minoricensis* J.J. Rodr. | Malvaceae | 13 | Rocky | EN | Endemic | 4,855 |
| *Mauranthemum ebusitanum* (Vogt) N. Torres & Rosselló | Asteraceae | 8 | Scrub | EN | Endemic | 3,366 |
| *Medicago citrina* (Font Quer) Greuter | Fabaceae | 11 | Littoral | EN | Subendemic | 3,913 |
| *Micromeria filiformis* (Aiton) Benth. | Lamiaceae | 554 | Rocky | LC | Subendemic | 2,800 |
| *Micromeria rodriguezii* Freyn & Janka | Lamiaceae | 220 | Scrub | LC | Endemic | 2,800 |
| *Naufraga balearica* Constance & Cannon | Apiaceae | 8 | Rocky | EN | Endemic | 3,830 |
| *Ononis crispa* L. subsp. *crispa* | Fabaceae | 18 | Scrub | LC | Endemic | 3,329 |
| *Ononis crispa* subsp. *zschakei* (F. Herm.) L. Sáez & Rosselló | Fabaceae | 10 | Scrub | EN | Endemic | 3,792 |
| *Ophrys bertolonii* subsp. *balearica* (Delforge) L. Sáez & Rosselló | Orchidaceae | 119 | Scrub | LC | Endemic | 4,735 |
| *Orobanche iamonnensis* Pujadas & Fraga | Orobanchaceae | 5 | Littoral | NT | Endemic | 3,849 |
| *Paeonia cambessedesii* (Willk.) Willk. | Paeoniaceae | 138 | Rocky | LC | Endemic | 4,831 |
| *Pastinaca lucida* L. | Apiaceae | 184 | Rocky | LC | Endemic | 3,209 |
| *Phlomis italica* L. | Lamiaceae | 228 | Scrub | LC | Endemic | 3,564 |
| *Polycarpon dunense* P. Fraga & Rosselló | Caryophyllaceae | 5 | Littoral | VU | Endemic | 3,756 |
| *Polycarpon polycarpoides* subsp. *colomense* (Porta) Pedrol | Caryophyllaceae | 82 | Littoral | LC | Endemic | 3,688 |
| *Polygonum romanum* subsp. *balearicum* Raffaelli & Villar | Polygonaceae | 20 | Stream | NT | Endemic | 4,292 |
| *Primula acaulis* subsp. *balearica* (Willk.) Greuter & Burdet | Primulaceae | 19 | Rocky | NT | Endemic | 4,382 |
| *Ranunculus paludosus* subsp. *barceloi* (Grau) L. Sáez, Rosselló & N. Torres | Ranunculaceae | 11 | Scrub | NT | Endemic | 3,968 |
| *Ranunculus weyleri* Willk. | Ranunculaceae | 10 | Rocky | VU | Endemic | 4,030 |
| *Rhamnus ludovici-salvatoris* R. Chodat | Rhamnaceae | 214 | Scrub | LC | Endemic | 4,051 |
| *Rhamnus oleoides* subsp. *bourgaeana* (Gand.) Rivas Mart. & J.M. Pizarro | Rhamnaceae | 111 | Scrub | LC | Endemic | 4,051 |
| *Romulea columnae* subsp. *assumptionis* (Font Quer) O. Bolòs, Vigo, Masalles & Ninot | Iridaceae | 139 | Scrub | LC | Subendemic | 4,040 |
| *Rubia balearica* (Willk.) Porta | Rubiaceae | 509 | Rocky | LC | Endemic | 3,158 |
| *Rubia caespitos*a (Font Quer & Marcos) Rosselló | Rubiaceae | 14 | Rocky | EN | Endemic | 3,622 |
| *Santolina magonica* (O. Bolòs, Molinier & P. Monts.) Romo | Asteraceae | 231 | Rocky | LC | Endemic | 2,824 |
| *Santolina vedranensis* (O. Bolòs & Vigo) L. Sáez, M. Serrano, S. Ortiz & R. Carbajal | Asteraceae | 1 | Rocky | CR | Endemic | 3,516 |
| *Scutellaria balearica* Barceló | Lamiaceae | 73 | Rocky | LC | Endemic | 3,564 |
| *Senecio varicosus* L. f. | Asteraceae | 180 | Littoral | LC | Endemic | 3,399 |
| *Sibthorpia africana* L. | Plantaginaceae | 345 | Rocky | LC | Endemic | 3,825 |
| *Silene cambessedesii* Boiss. & Reut. | Caryophyllaceae | 63 | Littoral | VU | Subendemic | 3,379 |
| *Silene hifacensis* Willk. | Caryophyllaceae | 25 | Rocky | VU | Subendemic | 3,629 |
| *Silene migjornensis* L. Sáez, Guasp, P.P. Ferrer, López-Alvarado & Rosselló | Caryophyllaceae | 5 | Littoral | EN | Endemic | 3,379 |
| *Silene mollissima* (L.) Pers. | Caryophyllaceae | 89 | Rocky | LC | Endemic | 3,165 |
| *Solenopsis balearica* (E. Wimm.) Aldasoro, Castrov., Sales & Hedge | Campanulaceae | 19 | Stream | NT | Endemic | 4,476 |
| *Sonchus willkommii* (Burnat & Barbey) Rosselló & L. Sáez | Asteraceae | 664 | Rocky | LC | Endemic | 3,016 |
| *Spiroceratium bicknellii* (Briq.) Wolff | Apiaceae | 37 | Rocky | NT | Endemic | 3,372 |
| *Taraxacum majoricense* Galán & L. Sáez | Asteraceae | 1 | Rocky | EN | Endemic | 3,560 |
| *Teucrium asiaticum* L. | Lamiaceae | 275 | Scrub | LC | Endemic | 2,944 |
| *Teucrium balearicum* (Pau) Castrov. & Bayon | Lamiaceae | 413 | Scrub | LC | Endemic | 2,944 |
| *Teucrium capitatum* subsp. *majoricum* (Rouy) T. Navarro & Rosua | Lamiaceae | 3636 | Scrub | LC | Endemic | 2,944 |
| *Teucrium cossonii* D. Wood subsp. *cossonii* | Lamiaceae | 71 | Rocky | LC | Endemic | 2,944 |
| *Teucrium cossonii* subp. *punicum* Mayol, Mus Rosselló & Torres | Lamiaceae | 12 | Rocky | EN | Endemic | 3,408 |
| *Thapsia gymnesica* Rosselló & Pujadas | Apiaceae | 139 | Rocky | LC | Endemic | 3,389 |
| *Thymelaea velutina* (Cambess.) Endl. | Thymelaeaceae | 87 | Littoral | LC | Endemic | 3,955 |
| *Thymus herba-barona* subsp. *bivalens* Mayol, L. Sáez & Rosselló | Lamiaceae | 2 | Scrub | EN | Endemic | 3,375 |
| *Thymus richardii* Pers. subsp. *richardii* | Lamiaceae | 20 | Rocky | VU | Endemic | 2,980 |
| *Thymus richardii* subsp. *ebusitanus* (Font Quer) Jalas | Lamiaceae | 12 | Rocky | VU | Endemic | 2,980 |
| *Urtica atrovirens* subsp. *bianorii* (Knoche) Font Quer & Garcias Font | Urticaceae | 23 | Rocky | NT | Endemic | 4,577 |
| *Vicia bifoliolata* J.J. Rodr. | Fabaceae | 11 | Scrub | EN | Endemic | 3,956 |
| *Viola jaubertiana* Marès & Vigin. | Violaceae | 80 | Rocky | NT | Endemic | 4,123 |
| *Viola stolonifera* J.J. Rodr. | Violaceae | 5 | Rocky | VU | Endemic | 4,185 |

**Table S2**: Species supposedly endemic to the Balearic Islands accepted in “Flora iberica” or recently described, whose taxonomic value is uncertain.

| **Taxon** | **Comments** |
| --- | --- |
| *Daucus carota* subsp. *majoricus* A. Pujadas | Reduced to synonymy of *D. carota* subsp. *hispanicus* (Gouan) Thell. by Martínez Flores et al. (2020) [113]. |
| *Limonium alcudianum* Erben | Provisionally included within *Limonium camposanum* Erben |
| *Limonium artruchium* Erben | Its taxonomic value is uncertain; it is morphologically related to *L. minoricense* and *L. virgatum.* |
| *Limonium balearicum* (Pignatti) Brullo | This species is included within *L. minutum*. The morphological variation and the phenotypic plasticity exhibited by *L. minutum* is highly remarkable, and on this basis several taxonomic entities were described; however, their taxonomic status is unclear. |
| *Limonium bonafei* Erben | Provisionally included within a broad concept of *L. fontqueri*. The type material of *L. bonafei* is a plant with rather short scapes and relatively few sterile branches, but there are other specimens in Cala Algar and its surroundings [identified by Erben (1993) [112] as *L. bonafei* x *L. virgatum*) with longer stems and numerous sterile branches, and which correspond to Majorcan populations assimilated to *L. fontqueri*. |
| *Limonium* *caprariense* (Font Quer & Marcos) Pignatti | This species is included within *L. minutum*. See comments under *L. balearicum*. |
| *Limonium carregadorense* Erben | This species is included within *L. pseudodyctiocladon*. On the other hand, some specimens referred to *L. carregadorense* could correspond to hybrids between *pseudodyctiocladon* and *L. virgatum*. |
| *Limonium connivens* Erben | Provisionally included under *Limonium companyonis* (Gren. & Billot) Kuntze |
| *Limonium* *dragonericum* Erben | This species is included within *L. minutum*. See comments under *L. balearicum*. |
| *Limonium* *ebusitanum* Font Quer | This species is included within *L. minutum*. See comments under *L. balearicum*. |
| *Limonium gymnesicum* Erben | Included under *L. majoricum* Pignatti |
| *Limonium* *muradense* Erben | This species is included within *L. minutum*. See comments under *L. balearicum*. |
| *Limonium orellii* Erben | This taxon, belonging to *L. companyonis* aggregate, is provisionally included under *L. bianorii* here. |
| *Limonium* *portopetranum* Erben | This species is included within *L. minutum*. See comments under *L. balearicum*. |
| *Limonium pseudebusitanum* Erben | This species is included within *L. minutum*. See comments under *L. balearicum*. |
| *Limonium pseudoarticulatum* Erben | This species is included within *L. pseudodyctiocladon*. |
| *Limonium retusum* L. Llorens | No clear morphological discontinuities exist within *L. formenterae* and *L. retusum*; these two taxa are probably conspecific. |
| *Limonium scorpioides* Erben | This species is included within *L. minutum*. See comments under *L. balearicum*. |
| *Limonium tamarindanum* Erben | Provisionally included under *Limonium virgatum* (Willd.) Fourr*.* |
| *Limonium* *tenuicaule* Erben. | This species is included within *L. minutum*. See comments under *L. balearicum*. |
| *Limonium validum* Erben | Provisionally included under *Limonium companyonis* (Gren. & Billot) Kuntze |
| Ophrys decembris S. Moingeon & J.-M. Moingeon | This species is included within *O. fusca* Link |
| *Ophrys fabrella* Delforge | This small-flowered plant is provisionally included under *O. bilunulata* Risso |
| *Orobanche rumseyana* A. Pujadas & P. Fraga | This name is considered a taxonomic synonym of *Phelipanche rosmarina* (Beck) Banfi, Galasso & Soldano [*Orobanche rosmarina* Beck] (see Sánchez Pedraja et al., 2023) [114]. |
| *Oxalis ferae* L. Llorens, L. Gil & C. Cardona | A taxon belonging to *Oxalis* sect. *Corniculatae* of uncertain taxonomic value. In "Flora iberica" this species was reduced to synonymy of *Oxalis filiformis* Kunth; however, a detailed study is required in order to elucidate its taxonomic position. |

**Table S3**: List of the compiled occurrence records of the endemic vascular flora of the Balearic Islands. (excel file)

**Table S4**: Changes in the assignment of the IUCN (2012) categories of endemic taxa with respect to Sáez et al. (2017).

| Taxon | Assessment (Sáez et al., 2017) | Current assessment |
| --- | --- | --- |
| *Arenaria bolosii* | CR B1ab(iii)+2ab(iii) | VU D2  In absence of evidence regarding a continuous decrease in its population (the species has been monitored in the last 5 years), the “Bb” subcriterion [“B”: small range (EOO and/or AOO), “b”: continuing decline, observed, inferred or projected (IUCN, 2012)] cannot be applied. However, in view of the very restricted range (AOO and EOO = 8 Km^2^) and specificity for a fragile habitat, this species is assessed as VU under criterion D2 |
| *Helleborus lividus* | LC | NT  It has an area of occupancy (AOO) of c. 50 km², and an extent of occurrence (EOO) of c. 450 km² (excluding areas without potential habitat between N and W Mallorca, E Mallorca and Cabrera Island). Its rocky habitat currently is more or less well protected, but there are potential threats from mountaineering and habitat disturbances caused by feral goats. |
| *Laserpitium gallicum* subsp. *majoricum* | LC | NT  It has an area of occupancy (AOO) of c. 50 km², and an extent of occurrence (EOO) of c. 400 km² (excluding areas without potential habitat between N and W Mallorca and E Mallorca). It is therefore assessed as Near Threatened as it is close to qualifying as threatened under criteria B and D2. In most locations the number of reproductive specimens is low. On the other hand, predation by feral goats and habitat changes can not be ruled out in some locations. |
| *Limonium fontqueri* | DD | VU D2  Considering a broad concept of this species (see comments under *L. bonafei*) but excluding specimens of intermediate morphology with *L. virgatum*, based on its geographical restriction which makes it vulnerable to stochastic events, as well as human activities, this species is assessed as VU under criterion D2. |
| *Silene migjornensis* | VU B1ab(iii)+2ab(iii); D2  (listed as *Silene sericea*) | EN B1ab(iii)c(ii-iv) + 2ab(iii)c(ii-iv). See Sáez et al. (2019). |
| *Taraxacum majoricense* | CR B1ab(iii,iv)c(iv)+2ab(iii,iv)c(iv) | EN D  It is a very rare species with a strongly restricted area (1 location, AOO and EOO = 4 Km^2^). In absence of clear evidence regarding a continuous decrease in its population or extreme fluctuations in the number of mature individuals, the “Bb” subcriterion [“B”: small range (EOO and/or AOO), “b” and “c” (IUCN, 2012)] cannot be applied. However, in view of the small population size (c. 80 reproductive specimens), this species is assessed as EN under criterion D. |
| *Viola stolonifera* | EN B1ac(iv)+2ac(iv) | VU D2  The number of locations has increased since the last assessment. It has an area of occupancy (AOO) of 12 km², and an extent of occurrence (EOO) of c. 16 km². In absence of clear evidence regarding a continuous decrease in its population or extreme fluctuations in the number of mature individuals, the “Bb” subcriterion [“B”: small range (EOO and/or AOO), “b” and “c” (IUCN, 2012)] cannot be applied. However, in view of the very restricted range and specificity for a fragile habitat, this species is assessed as VU under criterion D2. |

**Table S5**: List of plant endemic species showing the total number of 1x1 km grid squares occupied by each species, the number of grid squares included in the top 1%, 2.5%, 5% and 10% hotspots and the percentage of the total grids included in each top 1%, 2.5%, 5% and 10% hotspots.

|  |  | Top 1% | | Top 2.5% | | Top 5% | | Top 10% | |
| --- | --- | --- | --- | --- | --- | --- | --- | --- | --- |
| Species | Total squares | Squares | %Included | Squares | %Included | Squares | %Included | Squares | %Included |
| *Agrostis barceloi* | 1 | 1 | 100,0 | 1 | 100,0 | 1 | 100,0 | 1 | 100,0 |
| *Aira minoricensis* | 5 | 5 | 100,0 | 5 | 100,0 | 5 | 100,0 | 5 | 100,0 |
| *Allium antonii-bolosii antonii-bolosii* | 50 | 10 | 20,0 | 16 | 32,0 | 29 | 58,0 | 44 | 88,0 |
| *Allium antonii-bolosii eivissanum* | 22 | 1 | 4,5 | 2 | 9,1 | 21 | 95,5 | 22 | 100,0 |
| *Allium grosii* | 19 | 2 | 10,5 | 6 | 31,6 | 17 | 89,5 | 17 | 89,5 |
| *Anthyllis hystrix* | 62 | 4 | 6,5 | 7 | 11,3 | 23 | 37,1 | 47 | 75,8 |
| *Anthyllis vulneraria balearica* | 39 | 26 | 66,7 | 35 | 89,7 | 38 | 97,4 | 39 | 100,0 |
| *Arenaria bolosii* | 3 | 3 | 100,0 | 3 | 100,0 | 3 | 100,0 | 3 | 100,0 |
| *Arenaria grandiflora glabrescens* | 22 | 20 | 90,9 | 21 | 95,5 | 21 | 95,5 | 22 | 100,0 |
| *Aristolochia bianorii* | 72 | 15 | 20,8 | 34 | 47,2 | 47 | 65,3 | 63 | 87,5 |
| *Arum pictum sagittifolium* | 391 | 85 | 21,7 | 166 | 42,5 | 231 | 59,1 | 302 | 77,2 |
| *Asperula paui* | 31 | 10 | 32,3 | 16 | 51,6 | 18 | 58,1 | 21 | 67,7 |
| *Asplenium majoricum* | 42 | 13 | 31,0 | 16 | 38,1 | 19 | 45,2 | 37 | 88,1 |
| *Astragalus balearicus* | 363 | 73 | 20,1 | 142 | 39,1 | 232 | 63,9 | 301 | 82,9 |
| *Avellinia longiaristata* | 3 | 3 | 100,0 | 3 | 100,0 | 3 | 100,0 | 3 | 100,0 |
| *Bellium artrutxensis* | 4 | 4 | 100,0 | 4 | 100,0 | 4 | 100,0 | 4 | 100,0 |
| *Beta maritima marcosii* | 9 | 1 | 11,1 | 3 | 33,3 | 3 | 33,3 | 3 | 33,3 |
| *Biscutella ebusitana* | 27 | 9 | 33,3 | 14 | 51,9 | 16 | 59,3 | 19 | 70,4 |
| *Brassica balearica* | 139 | 64 | 46,0 | 105 | 75,5 | 125 | 89,9 | 134 | 96,4 |
| *Brimeura duvigneaudii duvigneaudii* | 9 | 0 | 0,0 | 2 | 22,2 | 9 | 100,0 | 9 | 100,0 |
| *Brimeura duvigneaudii occultata* | 1 | 1 | 100,0 | 1 | 100,0 | 1 | 100,0 | 1 | 100,0 |
| *Bupleurum barceloi* | 132 | 60 | 45,5 | 97 | 73,5 | 119 | 90,2 | 127 | 96,2 |
| *Carduncellus dianius* | 13 | 5 | 38,5 | 8 | 61,5 | 8 | 61,5 | 8 | 61,5 |
| *Carduus ibizensis* | 12 | 4 | 33,3 | 10 | 83,3 | 12 | 100,0 | 12 | 100,0 |
| *Carex rorulenta* | 302 | 90 | 29,8 | 172 | 57,0 | 230 | 76,2 | 274 | 90,7 |
| *Cephalaria squamiflora balearica* | 145 | 66 | 45,5 | 108 | 74,5 | 130 | 89,7 | 135 | 93,1 |
| *Cephalaria squamiflora ebusitana* | 3 | 3 | 100,0 | 3 | 100,0 | 3 | 100,0 | 3 | 100,0 |
| *Chaenorhinum formenterae* | 28 | 2 | 7,1 | 8 | 28,6 | 20 | 71,4 | 27 | 96,4 |
| *Chaenorhinum rodriguezii* | 6 | 6 | 100,0 | 6 | 100,0 | 6 | 100,0 | 6 | 100,0 |
| *Clinopodium rouyanum* | 11 | 10 | 90,9 | 11 | 100,0 | 11 | 100,0 | 11 | 100,0 |
| *Coristospermum huteri* | 3 | 3 | 100,0 | 3 | 100,0 | 3 | 100,0 | 3 | 100,0 |
| *Coronilla montserratii* | 10 | 3 | 30,0 | 8 | 80,0 | 10 | 100,0 | 10 | 100,0 |
| *Cotoneaster majoricensis* | 3 | 3 | 100,0 | 3 | 100,0 | 3 | 100,0 | 3 | 100,0 |
| *Crepis triasii* | 427 | 105 | 24,6 | 201 | 47,1 | 300 | 70,3 | 372 | 87,1 |
| *Crocus cambessedesii* | 444 | 103 | 23,2 | 193 | 43,5 | 289 | 65,1 | 376 | 84,7 |
| *Cyclamen balearicum* | 788 | 131 | 16,6 | 256 | 32,5 | 388 | 49,2 | 537 | 68,1 |
| *Cymbalaria fragilis* | 15 | 2 | 13,3 | 7 | 46,7 | 11 | 73,3 | 14 | 93,3 |
| *Dactylis ibicensis* | 8 | 3 | 37,5 | 6 | 75,0 | 7 | 87,5 | 7 | 87,5 |
| *Daphne rodriguezii* | 40 | 2 | 5,0 | 4 | 10,0 | 19 | 47,5 | 38 | 95,0 |
| *Delphinium pentagynum formenteranum* | 12 | 0 | 0,0 | 7 | 58,3 | 11 | 91,7 | 11 | 91,7 |
| *Dianthus rupicola bocchoriana* | 13 | 4 | 30,8 | 7 | 53,8 | 8 | 61,5 | 9 | 69,2 |
| *Digitalis minor* | 350 | 89 | 25,4 | 169 | 48,3 | 231 | 66,0 | 287 | 82,0 |
| *Diplotaxis ibicensis* | 78 | 9 | 11,5 | 19 | 24,4 | 29 | 37,2 | 49 | 62,8 |
| *Dryopteris pallida balearica* | 85 | 55 | 64,7 | 75 | 88,2 | 80 | 94,1 | 84 | 98,8 |
| *Erodium reichardii* | 59 | 24 | 40,7 | 39 | 66,1 | 47 | 79,7 | 53 | 89,8 |
| *Euphorbia fontqueriana* | 2 | 2 | 100,0 | 2 | 100,0 | 2 | 100,0 | 2 | 100,0 |
| *Euphorbia maresii balearica* | 16 | 12 | 75,0 | 14 | 87,5 | 14 | 87,5 | 16 | 100,0 |
| *Euphorbia maresii maresii* | 70 | 10 | 14,3 | 24 | 34,3 | 41 | 58,6 | 63 | 90,0 |
| *Euphorbia margalidiana* | 1 | 1 | 100,0 | 1 | 100,0 | 1 | 100,0 | 1 | 100,0 |
| *Euphorbia nurae* | 25 | 5 | 20,0 | 9 | 36,0 | 15 | 60,0 | 23 | 92,0 |
| *Femeniasia balearica* | 9 | 1 | 11,1 | 4 | 44,4 | 8 | 88,9 | 8 | 88,9 |
| *Filago petro-ianii* | 4 | 4 | 100,0 | 4 | 100,0 | 4 | 100,0 | 4 | 100,0 |
| *Galium balearicum* | 47 | 40 | 85,1 | 45 | 95,7 | 46 | 97,9 | 46 | 97,9 |
| *Galium crespianum* | 271 | 92 | 33,9 | 165 | 60,9 | 216 | 79,7 | 244 | 90,0 |
| *Galium friedrichii* | 41 | 10 | 24,4 | 18 | 43,9 | 24 | 58,5 | 33 | 80,5 |
| *Genista dorycnifolia dorycnifolia* | 25 | 1 | 4,0 | 3 | 12,0 | 20 | 80,0 | 23 | 92,0 |
| *Genista dorycnifolia grosii* | 10 | 7 | 70,0 | 9 | 90,0 | 9 | 90,0 | 9 | 90,0 |
| *Genista majorica* | 266 | 78 | 29,3 | 147 | 55,3 | 205 | 77,1 | 240 | 90,2 |
| *Genista valdes-bermejoi* | 20 | 13 | 65,0 | 18 | 90,0 | 19 | 95,0 | 20 | 100,0 |
| *Globularia majoricensis* | 207 | 74 | 35,7 | 129 | 62,3 | 166 | 80,2 | 188 | 90,8 |
| *Helianthemum scopulicolum* | 1 | 1 | 100,0 | 1 | 100,0 | 1 | 100,0 | 1 | 100,0 |
| *Helichrysum crassifolium* | 489 | 106 | 21,7 | 204 | 41,7 | 301 | 61,6 | 386 | 78,9 |
| *Helichrysum massanellanum* | 13 | 9 | 69,2 | 11 | 84,6 | 13 | 100,0 | 13 | 100,0 |
| *Helictotrichon crassifolium* | 13 | 8 | 61,5 | 12 | 92,3 | 12 | 92,3 | 12 | 92,3 |
| *Helosciadium bermejoi* | 1 | 1 | 100,0 | 1 | 100,0 | 1 | 100,0 | 1 | 100,0 |
| *Helleborus lividus* | 52 | 21 | 40,4 | 34 | 65,4 | 45 | 86,5 | 47 | 90,4 |
| *Hieracium balearicum* | 17 | 17 | 100,0 | 17 | 100,0 | 17 | 100,0 | 17 | 100,0 |
| *Hieracium majoricanum* | 6 | 6 | 100,0 | 6 | 100,0 | 6 | 100,0 | 6 | 100,0 |
| *Hippocrepis balearica* | 486 | 103 | 21,2 | 192 | 39,5 | 280 | 57,6 | 365 | 75,1 |
| *Hippocrepis grosii* | 17 | 6 | 35,3 | 8 | 47,1 | 9 | 52,9 | 9 | 52,9 |
| *Hypericum balearicum* | 535 | 103 | 19,3 | 202 | 37,8 | 321 | 60,0 | 422 | 78,9 |
| *Hypericum hircinum cambessedesii* | 16 | 9 | 56,3 | 14 | 87,5 | 16 | 100,0 | 16 | 100,0 |
| *Laserpitium gallicum majoricum* | 56 | 42 | 75,0 | 51 | 91,1 | 54 | 96,4 | 55 | 98,2 |
| *Launaea cervicornis* | 340 | 30 | 8,8 | 64 | 18,8 | 115 | 33,8 | 200 | 58,8 |
| *Limonium alcudianum* | 7 | 4 | 57,1 | 6 | 85,7 | 6 | 85,7 | 6 | 85,7 |
| *Limonium antoni-llorensii* | 4 | 4 | 100,0 | 4 | 100,0 | 4 | 100,0 | 4 | 100,0 |
| *Limonium barceloi* | 1 | 1 | 100,0 | 1 | 100,0 | 1 | 100,0 | 1 | 100,0 |
| *Limonium bianorii* | 7 | 0 | 0,0 | 2 | 28,6 | 3 | 42,9 | 3 | 42,9 |
| *Limonium biflorum* | 40 | 10 | 25,0 | 11 | 27,5 | 16 | 40,0 | 35 | 87,5 |
| *Limonium boirae* | 2 | 2 | 100,0 | 2 | 100,0 | 2 | 100,0 | 2 | 100,0 |
| *Limonium bolosii* | 1 | 1 | 100,0 | 1 | 100,0 | 1 | 100,0 | 1 | 100,0 |
| *Limonium carvalhoi* | 1 | 1 | 100,0 | 1 | 100,0 | 1 | 100,0 | 1 | 100,0 |
| *Limonium ejulabilis* | 1 | 1 | 100,0 | 1 | 100,0 | 1 | 100,0 | 1 | 100,0 |
| *Limonium escarrei* | 1 | 1 | 100,0 | 1 | 100,0 | 1 | 100,0 | 1 | 100,0 |
| *Limonium fontqueri* | 21 | 4 | 19,0 | 7 | 33,3 | 20 | 95,2 | 21 | 100,0 |
| *Limonium formenterae* | 9 | 1 | 11,1 | 6 | 66,7 | 7 | 77,8 | 7 | 77,8 |
| *Limonium grosii* | 24 | 1 | 4,2 | 9 | 37,5 | 18 | 75,0 | 20 | 83,3 |
| *Limonium inexpectans* | 2 | 2 | 100,0 | 2 | 100,0 | 2 | 100,0 | 2 | 100,0 |
| *Limonium leonardi-llorensii* | 2 | 2 | 100,0 | 2 | 100,0 | 2 | 100,0 | 2 | 100,0 |
| *Limonium magallufianum* | 2 | 2 | 100,0 | 2 | 100,0 | 2 | 100,0 | 2 | 100,0 |
| *Limonium majoricum* | 4 | 1 | 25,0 | 3 | 75,0 | 3 | 75,0 | 3 | 75,0 |
| *Limonium marisoliis* | 3 | 2 | 66,7 | 3 | 100,0 | 3 | 100,0 | 3 | 100,0 |
| *Limonium migjornense* | 3 | 3 | 100,0 | 3 | 100,0 | 3 | 100,0 | 3 | 100,0 |
| *Limonium minoricense* | 9 | 1 | 11,1 | 3 | 33,3 | 9 | 100,0 | 9 | 100,0 |
| *Limonium minutum* | 660 | 47 | 7,1 | 108 | 16,4 | 191 | 28,9 | 302 | 45,8 |
| *Limonium pseudodyctiocladon* | 1 | 1 | 100,0 | 1 | 100,0 | 1 | 100,0 | 1 | 100,0 |
| *Limonium saxicolum* | 4 | 1 | 25,0 | 3 | 75,0 | 3 | 75,0 | 3 | 75,0 |
| *Limonium wiedmannii* | 3 | 1 | 33,3 | 1 | 33,3 | 1 | 33,3 | 1 | 33,3 |
| *Linaria aeruginea pruinosa* | 92 | 51 | 55,4 | 74 | 80,4 | 83 | 90,2 | 88 | 95,7 |
| *Lonicera pyrenaica majoricensis* | 34 | 28 | 82,4 | 33 | 97,1 | 34 | 100,0 | 34 | 100,0 |
| *Lotus fulgurans* | 55 | 3 | 5,5 | 8 | 14,5 | 22 | 40,0 | 44 | 80,0 |
| *Lotus tetraphyllus* | 308 | 63 | 20,5 | 116 | 37,7 | 178 | 57,8 | 243 | 78,9 |
| *Magydaris pastinacea femeniesii* | 5 | 4 | 80,0 | 4 | 80,0 | 5 | 100,0 | 5 | 100,0 |
| *Malva minoricensis* | 13 | 2 | 15,4 | 6 | 46,2 | 10 | 76,9 | 13 | 100,0 |
| *Mauranthemum ebusitanum* | 8 | 4 | 50,0 | 8 | 100,0 | 8 | 100,0 | 8 | 100,0 |
| *Medicago citrina* | 11 | 1 | 9,1 | 5 | 45,5 | 7 | 63,6 | 7 | 63,6 |
| *Micromeria filiformis* | 554 | 104 | 18,8 | 196 | 35,4 | 299 | 54,0 | 394 | 71,1 |
| *Micromeria rodriguezii* | 220 | 28 | 12,7 | 45 | 20,5 | 68 | 30,9 | 109 | 49,5 |
| *Naufraga balearica* | 8 | 2 | 25,0 | 5 | 62,5 | 6 | 75,0 | 6 | 75,0 |
| *Ononis crispa crispa* | 18 | 4 | 22,2 | 7 | 38,9 | 13 | 72,2 | 17 | 94,4 |
| *Ononis crispa zschakei* | 10 | 6 | 60,0 | 10 | 100,0 | 10 | 100,0 | 10 | 100,0 |
| *Ophrys bertolonii balearica* | 119 | 17 | 14,3 | 30 | 25,2 | 37 | 31,1 | 61 | 51,3 |
| *Orobanche iammonensis* | 5 | 0 | 0,0 | 4 | 80,0 | 4 | 80,0 | 4 | 80,0 |
| *Paeonia cambessedesii* | 138 | 39 | 28,3 | 59 | 42,8 | 74 | 53,6 | 108 | 78,3 |
| *Pastinaca lucida* | 184 | 69 | 37,5 | 116 | 63,0 | 147 | 79,9 | 171 | 92,9 |
| *Phlomis italica* | 228 | 78 | 34,2 | 135 | 59,2 | 168 | 73,7 | 201 | 88,2 |
| *Polycarpon dunense* | 5 | 2 | 40,0 | 5 | 100,0 | 5 | 100,0 | 5 | 100,0 |
| *Polycarpon polycarpoides colomense* | 82 | 12 | 14,6 | 27 | 32,9 | 45 | 54,9 | 63 | 76,8 |
| *Polygonum romanum balearicum* | 20 | 0 | 0,0 | 4 | 20,0 | 12 | 60,0 | 18 | 90,0 |
| *Primula acaulis balearica* | 19 | 16 | 84,2 | 18 | 94,7 | 19 | 100,0 | 19 | 100,0 |
| *Ranunculus paludosus barceloi* | 11 | 6 | 54,5 | 10 | 90,9 | 11 | 100,0 | 11 | 100,0 |
| *Ranunculus weyleri* | 10 | 7 | 70,0 | 9 | 90,0 | 10 | 100,0 | 10 | 100,0 |
| *Rhamnus ludovici-salvatoris* | 214 | 42 | 19,6 | 88 | 41,1 | 151 | 70,6 | 193 | 90,2 |
| *Rhamnus oleoides bourgeana* | 111 | 43 | 38,7 | 72 | 64,9 | 88 | 79,3 | 103 | 92,8 |
| *Romulea columnae assumptionis* | 139 | 21 | 15,1 | 43 | 30,9 | 67 | 48,2 | 103 | 74,1 |
| *Rubia balearica* | 509 | 111 | 21,8 | 218 | 42,8 | 337 | 66,2 | 439 | 86,2 |
| *Rubia caespitosa* | 14 | 2 | 14,3 | 4 | 28,6 | 11 | 78,6 | 11 | 78,6 |
| *Santolina magonica* | 231 | 66 | 28,6 | 107 | 46,3 | 140 | 60,6 | 189 | 81,8 |
| *Santolina vedranensis* | 1 | 1 | 100,0 | 1 | 100,0 | 1 | 100,0 | 1 | 100,0 |
| *Scutellaria balearica* | 73 | 45 | 61,6 | 58 | 79,5 | 65 | 89,0 | 71 | 97,3 |
| *Senecio varicosus* | 180 | 20 | 11,1 | 52 | 28,9 | 88 | 48,9 | 138 | 76,7 |
| *Sibthorpia africana* | 345 | 96 | 27,8 | 180 | 52,2 | 261 | 75,7 | 311 | 90,1 |
| *Silene cambessedesii* | 63 | 1 | 1,6 | 9 | 14,3 | 21 | 33,3 | 35 | 55,6 |
| *Silene hifacensis* | 25 | 13 | 52,0 | 21 | 84,0 | 21 | 84,0 | 21 | 84,0 |
| *Silene migjornensis* | 5 | 5 | 100,0 | 5 | 100,0 | 5 | 100,0 | 5 | 100,0 |
| *Silene mollissima* | 89 | 45 | 50,6 | 64 | 71,9 | 74 | 83,1 | 82 | 92,1 |
| *Solenopsis balearica* | 19 | 7 | 36,8 | 12 | 63,2 | 15 | 78,9 | 17 | 89,5 |
| *Sonchus willkommii* | 664 | 122 | 18,4 | 235 | 35,4 | 365 | 55,0 | 491 | 73,9 |
| *Spiroceratium bicknellii* | 37 | 8 | 21,6 | 25 | 67,6 | 35 | 94,6 | 35 | 94,6 |
| *Taraxacum majoricense* | 1 | 1 | 100,0 | 1 | 100,0 | 1 | 100,0 | 1 | 100,0 |
| *Teucrium asiaticum* | 275 | 81 | 29,5 | 157 | 57,1 | 221 | 80,4 | 259 | 94,2 |
| *Teucrium balearicum* | 413 | 110 | 26,6 | 200 | 48,4 | 286 | 69,2 | 362 | 87,7 |
| *Teucrium capitatum majoricum* | 3636 | 219 | 6,0 | 399 | 11,0 | 668 | 18,4 | 1046 | 28,8 |
| *Teucrium cossonii cossonii* | 71 | 30 | 42,3 | 45 | 63,4 | 55 | 77,5 | 60 | 84,5 |
| *Teucrium cossonii punicum* | 12 | 7 | 58,3 | 10 | 83,3 | 10 | 83,3 | 10 | 83,3 |
| *Thapsia gymnesica* | 139 | 23 | 16,5 | 41 | 29,5 | 68 | 48,9 | 101 | 72,7 |
| *Thymelaea velutina* | 87 | 28 | 32,2 | 39 | 44,8 | 52 | 59,8 | 73 | 83,9 |
| *Thymus herba-barona bivalens* | 2 | 2 | 100,0 | 2 | 100,0 | 2 | 100,0 | 2 | 100,0 |
| *Thymus richardii ebusitanus* | 20 | 10 | 50,0 | 15 | 75,0 | 16 | 80,0 | 16 | 80,0 |
| *Thymus richardii richardii* | 12 | 9 | 75,0 | 11 | 91,7 | 11 | 91,7 | 11 | 91,7 |
| *Urtica atrovirens bianorii* | 23 | 9 | 39,1 | 17 | 73,9 | 21 | 91,3 | 22 | 95,7 |
| *Vicia bifoliolata* | 11 | 3 | 27,3 | 5 | 45,5 | 10 | 90,9 | 11 | 100,0 |
| *Viola jaubertiana* | 80 | 25 | 31,3 | 53 | 66,3 | 68 | 85,0 | 76 | 95,0 |
| *Viola stolonifera* | 5 | 2 | 40,0 | 5 | 100,0 | 5 | 100,0 | 5 | 100,0 |

**Supplementary References**

112. Erben, M. 1993. Limonium Mill. In Castroviejo, S., C. Aedo, S. Cirujano, M. Laínz, P. Montserrat, R. Morales, F. Muñoz Garmendia, C. Navarro, J. Paiva & C. Soriano (eds.). Flora iberica III. Plumbaginaceae (partim)-Capparaceae: 2-143. Real Jardín Botánico, CSIC. Madrid.

113. Martínez Flores, F., M.B. Crespo, Ph. Simon, H. Ruess, K. Reitsma, E. Geoffriau, Ch. Allender, N. Mezghani & D.M. Spooner (2020). Subspecies Variation of Daucus carota Coastal (“Gummifer”) Morphotypes (Apiaceae) Using Genotyping-by-Sequencing. Syst. Bot. 45: 688-702.

114. Sánchez Pedraja, Ó., G. Moreno Moral, L. Carlón, R. Piwowarczyk, M. Laínz & G.M. Schneeweiss 2016 [continuously updated]. Index of Orobanchaceae. http://www.farmalierganes.com/Otrospdf/publica/Orobanchaceae%20Index.htm. Liérganes, Cantabria, Spain. ISSN: 2386-9666 (accessed, 20 May 2023)
