# Supplementary material for: Are Mediterranean Island Mountains Hotspots of Taxonomic and Phylogenetic Biodiversity? The Case of the Endemic Flora of the Balearic Islands
Source: Plants (Basel). 2023 Jul 13;12(14):2640. doi: 10.3390/plants12142640 (PMC10386412; doi:10.3390/plants12142640)
Supplement: Supplementary file 1 [file plants-12-02640-s001.zip › plants-2484991-supplementary/Phylogeny_Endemics_Balearic_Islands.pdf]

((((((((((((((Helichrysum_crassifolium:0.129388,Helichrysum_massanellanum:0.129388):6.6288
63,Filago_petroianii:6.75825):16.231209,((Bellium_artrutxensis:18.943101,(Mauranthemum_e
busitanum:4.203878,(Santolina_vedranensis:1.462724,Santolina_magonica:1.462724):2.74115
4):14.739223):2.505677,Senecio_varicosus:21.448778):1.540681):7.684679,(((Crepis_triasii:10
.582349,Taraxacum_majoricense:10.582348):1.958913,(Sonchus_willkommii:7.150311,Launae
a_cervicornis:7.150311):5.39095):4.033222,(Hieracium_majoricanum:2.627488,Hieracium_bal
earicum:2.627488):13.946995):14.099655):3.606085,((Carduncellus_dianius:4.084117,Femeni
asia_balearica:4.084118):8.045976,Carduus_ibicensis:12.130093):22.15013):48.922542,Soleno
psis_balearica:83.202765):10.527446,((Helosciadium_bermejoi:55.97716,(((((Coristospermum
_huteri:9.372858,Pastinaca_lucida:9.372858):0.01924,Magydaris_pastinacea_subsp_femeniesi
i:9.392097):7.110222,(Spiroceratium_bicknellii:15.428434,Naufraga_balearica:15.428435):1.07
3885):6.030024,(Thapsia_gymnesica:13.116344,Laserpitium_gallicum_subsp_majoricum:13.11
6343):9.416):8.940589,Bupleurum_barceloi:31.472933):24.504228):29.663494,((Cephalaria_s
quamiflora_subsp_ebusitana:9.561829,Cephalaria_squamiflora_subsp_balearica:9.561829):41.
189844,Lonicera_pyrenaica_subsp_majoricensis:50.751673):34.888981):8.089557):13.011199,
(((((Clinopodium_rouyanum:9.562142,((Thymus_richardii_subsp_richardii:6.483165,Thymus_ri
chardii_subsp_ebusitanus:6.483165,Thymus_herba_barona_subsp_bivalens:6.483165):2.7663
28,(Micromeria_rodriguezii:1.689957,Micromeria_filiformis:1.689957):7.559536):0.312649):24
.718275,((Teucrium_balearicum:7.091961,Teucrium_cossonii_subsp_punicum:7.091961,Teucri
um_cossonii_subsp_cossonii:7.091961,Teucrium_capitatum_subsp_majoricum:7.091961,Teucri
um_asiaticum:7.091962):21.483752,(Phlomis_italica:26.819148,Scutellaria_balearica:26.81914
8):1.756565):5.704704):6.008518,Orobanche_iammonensis:40.288935):12.643826,((Digitalis_
minor:37.776553,(Globularia_majoricensis:37.30874,Sibthorpia_africana:37.30874):0.467812):
3.587635,((Chaenorhinum_rodriguezii:23.403655,Chaenorhinum_formenterae:23.403655,Lina
ria_aeruginea_subsp_pruinosa:23.403655):1.478595,Cymbalaria_fragilis:24.88225):16.481937
):11.568574):36.818306,(((Galium_crespianum:6.40221,Galium_balearicum:6.40221,Galium_fr
iedrichii:6.402211):0.465838,Asperula_paui:6.868049):1.32096,(Rubia_caespitosa:4.313858,R
ubia_balearica:4.313858):3.87515):81.562059):16.990343):5.599318,(Primula_acaulis_subsp_
balearica:40.606129,Cyclamen_balearicum:40.60613):71.734599):7.533505,((((((Silene_cambe
ssedesii:7.727341,(Silene_mollissima:0.355087,Silene_hifacensis:0.355087):7.372255,Silene_
migjornensis:7.727342):35.040483,Dianthus_rupicola_subsp_bocchoriana:42.767824):8.05200
7,(Arenaria_grandiflora_subsp_glabrescens:32.806215,Arenaria_bolosii:32.806215):18.013616
):8.0618,(Polycarpon_polycarpoides_subsp_colomense:4.737134,Polycarpon_dunense:4.7371
34):54.144497):11.231,Beta_maritima_subsp_marcosii:70.112631):36.938676,(Polygonum_ro
manum_subsp_balearicum:67.902135,(Limonium_pseudodyctiocladon:14.304962,Limonium_
migjornense:14.304962,Limonium_marisoliis:14.304962,Limonium_majoricum:14.304962,Lim
onium_leonardillorensii:14.304962,Limonium_grosii:14.304962,Limonium_formenterae:14.30
4962,Limonium_escarrei:14.304962,Limonium_carvalhoi:14.304962,Limonium_bolosii:14.304
962,Limonium_antonillorensii:14.304962,(((((Limonium_inexpectans:0.578632,((Limonium_sax
icolum:0.004305,Limonium_alcudianum:0.004305):0.003505,Limonium_boirae:0.00781):0.570
822):0.00076,((((Limonium_biflorum:0.008361,Limonium_fontqueri:0.008361):0.002903,Limo
nium_minoricense:0.011264):0.007207,Limonium_minutum:0.018471):0.009266,Limonium_bi
anorii:0.027737):0.551655):0.925774,Limonium_wiedmannii:1.505166):1.106417,Limonium_b
arceloi:2.611583):0.130553,(Limonium_ejulabilis:0.016007,Limonium_magallufianum:0.01600
7):2.726129):11.562827):53.597173):39.149172):12.822926):3.860003,((((((((Medicago_citrin
a:16.48758,(Ononis_crispa_subsp_zschakei:9.33178,Ononis_crispa_subsp_crispa:9.33178):7.1
558):2.071661,Vicia_bifoliolata:18.559241):13.700617,Astragalus_balearicus:32.259858):17.16
6662,((Lotus_tetraphyllus:18.992657,(Lotus_fulgurans:17.789769,(Anthyllis_vulneraria_subsp_

balearica:6.628814,Anthyllis_hystrix:6.628814):11.160955):1.202888):4.258862,(Coronilla_mo
ntserratii:20.863903,(Hippocrepis_grosii:4.159083,Hippocrepis_balearica:4.159083):16.70482):
2.387616):26.175001):14.789405,(Genista_valdesbermejoi:12.705955,Genista_majorica:12.70
5955,Genista_dorycnifolia_subsp_grosii:12.705955,Genista_dorycnifolia_subsp_dorycnifolia:1
2.705955):51.50997):48.48527,(Cotoneaster_majoricensis:98.961795,(Urtica_atrovirens_subsp
_bianorii:85.493415,(Rhamnus_oleoides_subsp_bourgeana:7.134798,Rhamnus_ludovicisalvat
oris:7.134798):78.358617):13.46838):13.7394):3.084369,(((Hypericum_hircinum_subsp_camb
essedesii:14.460606,Hypericum_balearicum:14.460607):80.404672,(Viola_stolonifera:14.9371
05,Viola_jaubertiana:14.937106):79.928173):7.74248,(Euphorbia_nurae:21.907575,Euphorbia
_margalidiana:21.907575,Euphorbia_maresii_subsp_maresii:21.907575,Euphorbia_maresii_su
bsp_balearica:21.907575,Euphorbia_fontqueriana:21.907576):80.700183):13.177806):2.79303
9,((((Brassica_balearica:22.378051,Diplotaxis_ibicensis:22.37805):4.652532,Biscutella_ebusita
na:27.030582):65.922554,((Malva_minoricensis:66.793042,Helianthemum_scopulicolum:66.7
93042):3.325043,(Thymelaea_velutina:9.714862,Daphne_rodriguezii:9.714862):60.403223):22
.835051):24.040059,Erodium_reichardii:116.993196):1.585408):3.826488,Paeonia_cambessed
esii:122.405092):1.329145):7.946463,(((Ranunculus_paludosus_subsp_barceloi:13.278987,Ran
unculus_weyleri:13.278988):27.265464,Helleborus_lividus:40.544452):1.241595,Delphinium_
pentagynum_subsp_formenteranum:41.786046):89.894653):4.077365,(((Ophrys_bertolonii_s
ubsp_balearica:108.958736,(((Brimeura_duvigneaudii_subsp_occultata:14.45216,Brimeura_du
vigneaudii_subsp_duvigneaudii:14.45216):48.041209,(Allium_grosii:24.753997,Allium_antonii
_bolosii_subsp_eivissanum:24.753997,Allium_antonii_bolosii_subsp_antonii_bolosii:24.75399
7):37.739372):18.164276,(Crocus_cambessedesii:15.081909,Romulea_columnae_subsp_assu
mptionis:15.081908):65.575737):28.301091):5.641826,((Avellinia_longiaristata:47.740111,((Da
ctylis_ibicensis:12.69896,Aira_minoricensis:12.698959):4.19433,(Agrostis_barceloi:13.161319,
Helictotrichon_crassifolium:13.161319):3.73197):30.846822):46.081395,Carex_rorulenta:93.82
1506):20.779056):16.198196,Arum_pictum_subsp_sagittifolium:130.798758):4.959306):0.154
122,Aristolochia_bianorii:135.912187):254.790353,(Dryopteris_pallida_subsp_balearica:90.63
4057,Asplenium_majoricum:90.634058):300.068482);
